# Quantitative Trait Locus Based Virulence Determinant Mapping of the HSV-1 Genome in Murine Ocular Infection: Genes Involved in Viral Regulatory and Innate Immune Networks Contribute to Virulence

**DOI:** 10.1371/journal.ppat.1005499

**Published:** 2016-03-10

**Authors:** Aaron W. Kolb, Kyubin Lee, Inna Larsen, Mark Craven, Curtis R. Brandt

**Affiliations:** 1 Department of Ophthalmology and Visual Sciences, University of Wisconsin-Madison, Madison, Wisconsin, United States of America; 2 Department of Computer Sciences, University of Wisconsin-Madison, Madison, Wisconsin, United States of America; 3 Department of Biostatistics and Medical Informatics, School of Medicine and Public Health, University of Wisconsin-Madison, Madison, Wisconsin, United States of America; 4 Department of Medical Microbiology and Immunology, School of Medicine and Public Health, University of Wisconsin-Madison, Madison, Wisconsin, United States of America; 5 McPherson Eye Research Institute, University of Wisconsin-Madison, Madison, Wisconsin, United States of America; Northwestern University, UNITED STATES

## Abstract

Herpes simplex virus type 1 causes mucocutaneous lesions, and is the leading cause of infectious blindness in the United States. Animal studies have shown that the severity of HSV-1 ocular disease is influenced by three main factors; innate immunity, host immune response and viral strain. We previously showed that mixed infection with two avirulent HSV-1 strains (OD4 and CJ994) resulted in recombinants that exhibit a range of disease phenotypes from severe to avirulent, suggesting epistatic interactions were involved. The goal of this study was to develop a quantitative trait locus (QTL) analysis of HSV-1 ocular virulence determinants and to identify virulence associated SNPs. Blepharitis and stromal keratitis quantitative scores were characterized for 40 OD4:CJ994 recombinants. Viral titers in the eye were also measured. Virulence quantitative trait locus mapping (vQTLmap) was performed using the Lasso, Random Forest, and Ridge regression methods to identify significant phenotypically meaningful regions for each ocular disease parameter. The most predictive Ridge regression model identified several phenotypically meaningful SNPs for blepharitis and stromal keratitis. Notably, phenotypically meaningful nonsynonymous variations were detected in the UL24, UL29 (ICP8), UL41 (VHS), UL53 (gK), UL54 (ICP27), UL56, ICP4, US1 (ICP22), US3 and gG genes. Network analysis revealed that many of these variations were in HSV-1 regulatory networks and viral genes that affect innate immunity. Several genes previously implicated in virulence were identified, validating this approach, while other genes were novel. Several novel polymorphisms were also identified in these genes. This approach provides a framework that will be useful for identifying virulence genes in other pathogenic viruses, as well as epistatic effects that affect HSV-1 ocular virulence.

## Author Summary

In addition to causing recurrent labial lesions, herpes simplex virus type 1 (HSV-1) is also the primary source of infectious blindness in the United States. Animal studies have shown that the severity of infection is influenced by several factors, including viral strain. Conventional studies investigating the genetics of viral virulence have focused on characterizing a naturally occurring strain, and engineering mutations into viruses. The purpose of this study was to develop a quantitative trait locus (QTL) computational analysis of HSV-1 genome to identify ocular virulence determinants and associated viral SNPs. Notably, phenotypically meaningful variations were detected in the UL24, UL29 (ICP8), UL41 (VHS), UL53 (gK), UL54 (ICP27), UL56, ICP4, US1 (ICP22), US3 and gG genes. Several genes previously implicated in virulence were identified, validating this approach, while other genes were novel. This is the first time a QTL based approach has been applied to a herpesvirus and it will also be valuable in future virulence, epistasis, and protein-protein interaction studies.

## Introduction

Herpes simplex virus type 1 (HSV-1) causes recurrent mucocutaneous lesions, is an increasing cause of genital herpes, and is the primary source of infectious blindness and sporadic encephalitis in the United States [1–4]. HSV-1 ocular infection in humans usually presents as conjunctivitis, which can then advance to epithelial keratitis or deeper corneal infection (stromal keratitis) [4]. HSV-1 viral replication in the corneal epithelium triggers cell death, and infiltration of CD4+ T-cells resulting in corneal damage [5]. Research using animal models has found that the severity of HSV-1 infections depends on three factors; innate immunity, host immune response, and viral strain [6–21]. Several groups have used gene mapping methods to identify *host* genes influencing the severity of HSV-1 ocular disease. Mapping studies in mice have identified loci on chromosomes 4, 5, 12, 13, and 14 associated with general clinical disease [22], as well as a locus near the TNF p55 receptor on the murine chromosome 6 associated with resistance to HSV-1 infection and reactivation [11]. Other studies have also discovered loci on mouse chromosome 16 associated with mortality [15], mouse chromosome 12 which was associated with weight loss [15], CD45 tyrosine phosphatase (protective immunity against encephalitis) [23], and the calcitonin receptor which was related to susceptibility to encephalitis [24]. Recently, the NK complex on the distal arm of murine chromosome 6 was found to restrict viral spread in the brains of C57BL/6 mice [25].

The severity of ocular disease has been shown to be influenced by the genetic makeup of viral strains [19, 26, 27] and conventional studies on the genetics of viral virulence have depended on isolating and characterizing a naturally occurring viral strain with altered virulence characteristics, genetically engineering either deletions or point mutations, or using marker transfer methods to exchange genes between strains.

It is highly likely that the virulence phenotype of a given strain of HSV is due to the effects of multiple genes and epistatic interactions between these genes. Early evidence for potential epistatic interactions was provided by Peter Wildy [28]. He carried out mixed infections with HSV strains using chorio-allantois membranes and isolated several recombinants that displayed altered plaque and virulence phenotypes. In 1986 Javier et al [29] showed that mixed infections with two avirulent HSV-1 strains generated lethal recombinant viruses. Subsequently, we [30] showed that mixed ocular infection with low virulence parental viruses generated recombinants with varying virulence phenotypes. More recently, the same phenomenon has been shown to occur in mixed genital infections [31].

In a previous study we used marker transfer of genomic segments from a moderately virulent strain, CJ394, into the avirulent strain OD4, to map genes associated with ocular and neurovirulence [32]. This study identified several virulence determinants, including UL41 (VHS), UL42 and US1 (ICP22), and suggested there was a complex interplay between viral genes since we obtained different disease phenotypes depending on which combinations of genes were transferred into the avirulent OD4 strain [6]. We also showed that two sequencing changes, S34A and Y116C in the ICP22 protein affected ocular virulence only when co-inherited [32, 33]. Our work was the first to actually identify genes involved in these epistatic interactions.

We have developed a novel approach incorporating QTL based analysis of the genomes and phenotypes of 40 HSV-1 viral recombinants generated by mixed infection with two avirulent parental strains; OD4 and CJ994 to identify genes affecting virulence. The blepharitis and stromal keratitis ocular disease phenotypes of each viral recombinant were quantified and combined with genomic sequence data for QTL based phenotypic association analysis. The QTL based analysis, which we term vQTLmap, used a Ridge regression model to identify several phenotypically meaningful features for blepharitis and stromal keratitis. Notably, amino-acid encoding nonsynonymous SNPs in UL24, ICP8, VHS, gK, UL56, ICP4, US1, US3, and gG were predicted to influence the severity of blepharitis. Nonsynonymous SNPs in VHS, ICP4, ICP22, US3 and gK were predicted to affect the severity of stromal keratitis. We also present evidence that the ability of the virus to replicate in the mouse is an important driver of virulence. The present study provides a methodology that will be useful for identifying additional virulence genes in HSV and other pathogenic viruses, examining epistatic interactions, and identifying protein-protein interaction networks that affect virulence.

## Materials and Methods

### Viruses

The recombinant viruses used in this study were generated by mixed infections with two avirulent parental strains; OD4 and CJ994. The OD4 and CJ994 strains were low passage clinically derived plaque purified isolates from Seattle, WA, USA, and the ocular disease phenotypes of the OD4 and CJ994 parental strains (blepharitis and stromal keratitis) were characterized previously [30]. The OD4-CJ994 recombinants were generated using both *in vivo* and *in vitro* methods [30, 34]. Briefly, the seventeen *in vivo* derived recombinants (Table 1) were derived as follows. Three to 4-week-old BALB/c female mice were infected by corneal scarification with 1 × 10^5^ PFU each of strain OD4 and CJ994. The corneas were removed at day 3 post-infection. homogenized, and freeze-thawed. The cleared lysates were serially diluted and titered on Vero (CCL-81, ATCC, Manassas, VA, USA) cells for plaque selection. The selected plaques were subjected to two additional rounds of plaque purification. Individual plaques (one per mouse to avoid isolating siblings) were picked and high-titer stocks were prepared in Vero cells as we have described previously [34].

**Table 1:** Strain derivation method, accession number, and sequence length of the OD4-CJ994 recombinants.

OD4-CJ994 Recombinant Name	Derivation Method	Accession Number	Sequence Length
**2-4-2**	*in vivo*	KR011288	152,245
**2-5-3**	*in vivo*	KR011292	152,249
**5-1-1**	*in vivo*	KR011299	152,242
**5-2-1**	*in vivo*	KR011285	152,197
**5-4-2**	*in vivo*	KR011311	152,259
**5-5-2**	*in vivo*	KR011295	152,238
**10-1-2**	*in vivo*	KR011302	152,263
**10-2-2**	*in vivo*	KR011277	152,240
**10-2-3**	*in vivo*	KR011274	152,236
**10-5-1**	*in vivo*	KR011301	152,238
**10-6-1**	*in vivo*	KR011296	152,227
**10-6-2**	*in vivo*	KR011306	152,236
**10-6-3**	*in vivo*	KR011284	152,185
**10-7-1**	*in vivo*	KR011290	152,248
**10-11-2**	*in vivo*	KR011287	152,256
**10-11-3**	*in vivo*	KR011309	152,233
**10-14-1**	*in vivo*	KR011291	152,235
**3M**	*in vitro*	KR011282	152,219
**4M**	*in vitro*	KR011278	152,172
**8S**	*in vitro*	KR011280	152,189
**11M**	*in vitro*	KR011294	152,140
**16S**	*in vitro*	KR011303	152,238
**19Lsyn**	*in vitro*	KR011293	152,122
**20L**	*in vitro*	KR011289	152,188
**26S**	*in vitro*	KR011308	152,207
**27S**	*in vitro*	KR011297	152,223
**31XL**	*in vitro*	KR011304	152,277
**34L**	*in vitro*	KR011275	152,279
**36L**	*in vitro*	KR011279	152,272
**47M**	*in vitro*	KR011305	152,298
**57M**	*in vitro*	KR011276	152,312
**65M**	*in vitro*	KR011312	152,178
**66M**	*in vitro*	KR011281	152,278
**76M**	*in vitro*	KR011300	152,249
**78S**	*in vitro*	KR052507	152,234
**81L**	*in vitro*	KR052508	152,263
**82S**	*in vitro*	KR011307	152,148
**83M**	*in vitro*	KR011310	152,232
**12-12-2**	*in vitro*	KR011298	152,288
**12-12-67**	*in vitro*	KR011286	152,276
**17**	Reference Strain	NC_001806	152,261
**OD4**	Parental Strain	JN420342	152,015
**CJ994**	Parental Strain	KR011283	152,233

The 23 *in vitro* derived recombinants (Table 1) were generated by infecting a 10cm plate of confluent Vero cells with 2 × 10^8^ PFU (multiplicity of infection, MOI, 10) of the OD4 and CJ994 (1:1 ratio) viruses. When the cells reached a 100% CPE, they were harvested and subjected to three freeze-thaw cycles. Clarified supernatants (400 xg, 10 minutes) were then serially diluted and plaqued onto Vero cells in six-well plates. Individual plaques were randomly picked, further plaque purified an additional two times, and high-titer stocks were prepared as described previously [34].

We deliberately chose to use two low virulence parents which were likely to generate recombinants with increases in virulence. This initial genetically restricted set of parents also increased the probability of finding significant associations with fewer recombinants.

### Ocular Disease Infection and Scoring

The ocular mouse model for HSV-1 disease used in this study has been described previously [26, 30, 35]. Briefly, for each OD4-CJ994 recombinant strain, the corneas of ten 4–6 week old Balb/C female mice were scarified using a 30 gauge hypodermic needle, and 5μL of medium (DMEM, 2% serum) containing 1 x 10^5^ pfu/ml of each recombinant virus was placed on the cornea. The mice were scored for blepharitis and stromal keratitis on days 1, 3, 5, 7, 9, 11, 13, and 15 post-infection. The scoring system was as follows. Blepharitis: 1 +, mild swelling of eyelids; 2+, moderate swelling with some crusting; 3 +, eye swollen 50% shut with severe crusting; 4 +, eye crusted shut. Stromal keratitis: 1 +, some haziness; iris detail visible; 2+, moderate clouding, iris detail obscured; 3+, cornea totally opaque; 4+, perforated cornea. Tear film samples were also taken on days 1, 3, 5, and 7 days post-infection, serially diluted, and then titered on Vero cell monolayers. The mean peak disease score (MPDS), which is the average of the most severe disease score for each mouse, per viral recombinant strain for the 15 day duration of the study was calculated for blepharitis and stromal keratitis. Mortality due to encephalitis was also recorded, but there weren’t enough neurovirulent strains to determine significant mortality associations. We also scored the severity of corneal neovascularization, but significant associations were not identified. This will require the analysis of additional recombinants.

### *In Vitro* Replication

Eighteen hour growth experiments were performed using both Vero cells and mouse embryonic fibroblasts (MEF; M-FB-481, Lonza, Walkersville, MD, USA). The Vero cells were cultured using Dulbecco’s Modified Eagle’s Medium (DMEM) plus 5% serum (1:1 mixture of fetal bovine serum and defined supplemented calf serum), and the MEF cells were cultured with DMEM containing 10% fetal bovine serum. The Vero and MEF cells were grown to confluency in 24-well plates in duplicate and infected with seven high pathogenic and nine low pathogenic OD4-CJ994 recombinants at a multiplicity of infection (MOI) of 1, with DMEM containing 2% serum. The cells were harvested 18 hours post-infection, centrifuged at 400 xg and subjected to three freeze-thaw cycles. The resulting cleared lysates (400 xg, 10 minutes) were then serially diluted and titered on Vero cells.

### Sequencing, Assembly and Multiple Sequence Alignment

The genomic sequencing and assembly of the 40 OD4-CJ994 HSV-1 recombinants has been previously described [34]. Briefly, twelve of the in vitro-derived recombinants were sequenced using an Illumina HiSeq 2000 sequencing system. One microgram of high-quality genomic DNA was submitted to the University of Wisconsin—Madison DNA Sequencing Facility for paired-end library preparation. Each library was generated using an Illumina TruSeq LT sample preparation kit (Illumina Inc., San Diego, CA, USA) per the manufacturer's specifications, with 300-bp fragments being targeted. Paired-end, 100-bp sequencing was performed in a single lane on the Illumina HiSeq 2000 sequencing system using SBS (version 3) kits, and an average of 5 million unique reads (1 Gb) was returned per library. FASTQ reports were created using the CASAVA (version 1.8.2) program.

The remaining recombinants were sequenced using the Illumina MiSeq platform, which produces longer reads. Five hundred nanograms of high-quality genomic DNA was submitted to the University of Wisconsin—Madison DNA Sequencing Facility for paired-end library preparation. Each library was generated using an Illumina TruSeq Nano LT sample preparation kit per the manufacturer's specifications, with 550-bp fragments being targeted. Paired-end, 250-bp sequencing was performed on the Illumina MiSeq platform using version 2 kits, and an average of 250,000 unique reads (125 Mb) was returned per library. FASTQ reports were created using the CASAVA (version 1.8.2) program.

The paired-end sequencing reads from the 40 recombinants and strain CJ994 were generated using a reference assembly. Between sequencing runs, an updated annotation was made available through GenBank; however, for consistency we decided to use the annotation with GenBank accession number NC_001806. The sequencing reads were aligned to the sequence of HSV-1 strain 17 using a local alignment method, with a consensus sequence subsequently being generated and extracted in a manner similar to that described previously [36]. Resulting gaps in the reference assembly were filled in with N′s (any nucleotide) without a proxy sequence, as has been done previously [37, 38]. The reason for not filling the sequencing gaps with proxy sequence is that these regions may not be phenotypically silent. Filling these regions with proxy sequence could produce false negatives in the QTL analysis. The genomes of the 40 OD4-CJ994 HSV-1 recombinants were aligned with the OD4 and CJ994 parental genomes using the MAFFT aligner from the SATé software package [39, 40]. The multiple sequence alignment is available for download at http://sites.ophth.wisc.edu/brandt/.

### Machine Learning vQTLmap Analysis

From the multiple sequence alignment (MSA), we first identified the alignment coordinates at which there were polymorphisms. We then filtered this set of MSA coordinates in order to limit our attention to those for which (i) the parental strains have different alleles, (ii) all recombinants have one of the two parental alleles, and (iii) the two parental alleles each occur in at least five of the recombinants. In order to reduce the effect of artifacts, such as large alignment gaps, we also filtered MSA coordinates that were in the neighborhood of at least 15 consecutive gaps. The motivation for filtering was to focus our analysis on the polymorphisms that occur frequently enough in our recombinant population that it would be possible to detect meaningful associations with the phenotypes. The potential downside of the filtering is that we might be excluding some functionally significant polymorphisms.

From the set of filtered MSA coordinates, we next aggregated neighboring coordinates into haplotype blocks. We defined a haplotype block as a contiguous region of the genome in which each recombinant has an identical pattern of inheritance across the SNPs. That is, for a given recombinant, all of the alleles within a haplotype block are either identical to the corresponding allele for one parent or the other. This process resulted in 491 haplotype blocks.

To form the representations for machine-learning algorithms, we defined a feature vector for each recombinant and parental strain such that there are two binary features per haplotype block and the values of the feature indicate whether that block was inherited from either the OD4 or CJ994 strain. The motivation for choosing a two-bit encoding per haplotype was to be able to represent other polymorphisms that are not inherited from either parental strain which will be included in future studies.

We applied three different machine-learning methods to learn models mapping the haplotype feature vectors described above to the three phenotypes of interest. We learned separate models for each phenotype where the phenotype for a given strain was the mean peak disease score for the animals infected with the strain. The learning methods we investigated were Random Forest regression [41], Lasso regression [42] and Ridge regression [43]. We selected these specific methods because they are all well suited to tasks with many irrelevant features, and because they represent two broad classes of models: whereas Lasso and Ridge learn linear functions, the Random-Forest approach learns a set of tree-based functions.

We used the R *randomForest* package (https://cran.r-project.org/web/packages/randomForest/) to learn Random-Forest models. The parameter specifying the number of trees in each forest was set to 1,000. All the other parameters of the algorithm were set to their default values. This decision was made in order to minimize the likelihood that the models would overfit the data set, given the small number of recombinants available. The random seed was set to the value 123 to ensure reproducibility in the random selection of bootstrap samples and the random selection of candidate features at each internal node in a tree.

To learn Lasso and Ridge regression models we used the R *glmnet* package (http://cran.r-project.org/web/packages/glmnet/). The Lasso or Ridge regression method was selected by setting the ‘alpha’ parameter value to 0 or 1 respectively. All other parameters were set to their default values. For each learned model, the λ parameter for both Lasso and Ridge was set to the value that minimized cross-validated error within each training set. 100 candidate values of λ considered during this process were determined by the *glmnet* package using its default procedure.

### Evaluation of Learned vQTLmap Models

In order to measure the extent to which the learned models were able to capture general relationships between viral genotypes and disease phenotypes, we used a leave-one-out cross-validation methodology. Specifically, on each iteration of the cross validation, we held aside one instance (consisting of a haplotype feature vector and the associated phenotype), learned a model using the remaining instances, and then applied the learned model to predict the phenotype of the held aside instance. We measured the accuracy of our predictions using both mean squared error (MSE) and R^2^ measures.

To assess the whether these measures showed statistically significant levels of predictive value, we employed a Monte Carlo methodology. For each phenotype, we repeated the cross-validation procedure 1,000 times with the measured phenotypes randomly assigned to the 42 haplotype feature vectors (i.e. the response variables in each data set were shuffled). For each randomized cross-validation, we measured the resulting R^2^ and MSE values as we did with the actual data. We constructed box plots to display the distribution of R^2^ and MSE values for the randomized data and to compare them to their counterpart values for the actual data.

### Identification of Genotype-Phenotype Associations

Since the Ridge regression models resulted in the best predictive accuracy among the three learning approaches, we focused on these models to identify the strongest genotype-phenotype associations. To do this, we used a Monte Carlo methodology that systematically considered each feature (i.e. haplotype block) in isolation and measured its impact on the MSE of the learned models. For each feature, we repeatedly created new test-set instances that were identical to the actual instances except that the values of the feature were permuted. That is, we retained the distribution of values for the given feature, but broke the dependence between the feature values and the phenotypes. We then measured the change in MSE when using the same cross-validation methodology as described above, but with the permuted feature. We did this 100 times for each feature, and then constructed Manhattan plots showing the average change in MSE as a function of genome coordinates (relative to HSV-1 strain 17). To determine thresholds for significance, we identified the largest *decrease* in MSE that occurred with the permuted data for each phenotype and negated this value. This approach was based on the assumption that decreases in MSE resulting from permuted data indicated variance due to noise and small sample sizes, and therefore meaningful associations were likely to show increases in MSE that had magnitudes at least as large as the largest decrease.

### HSV-1 Virulence Protein Interaction Network

A protein-protein interaction network based on vQTLmap identified proteins containing nonsynonymous variations was assembled using literature searches in conjunction with GADGET (http://gadget.biostat.wisc.edu/), a tool that finds and ranks genes that are associated with a concept of interest in the biomedical literature. The network was generated using Cytoscape 3.2.0 [44]. A full list of the protein-protein interactions assembled through literature searches is located in S3 Table.

### Linear Regression Scatter Plots and Statistical Analysis

Linear regression scatter plots of the mean peak titers versus mean peak disease scores for blepharitis and stromal keratitis were constructed using SigmaPlot 12.0 (Systat, San Jose, CA, USA). To determine statistical differences between the MPDS scores of *in vivo* and *in vitro* derived recombinants, non-parametric Mann-Whitney rank sum tests were performed using SigmaPlot 12.0. Mann-Whitney rank sum tests were also performed to establish if the mean peak tear film titers were statistically different between the *in vivo* and *in vitro* derived strains. To ascertain if trends in ocular disease associated with vQTLmap identified features were statistically significant, the mean peak disease scores for blepharitis and stromal keratitis for each of the 40 recombinants were first combined. For each notable vQTLmap feature, Mann-Whitney rank sum test was performed by placing the combined MPDS scores associated with SNPs derived from the OD4 parent in one array, and from the CJ994 parent in the other array.

### Ethics Statement

This research was approved by the University of Wisconsin-Madison School of Medicine and Public Health Institutional Animal Care and Use Committee (Animal Welfare Assurance Number A3368-01) as protocol # M00267, expiration date 5/20/2018. Our IACUC adheres to the guidelines mandated by the Animal Welfare act, administered by the United States Department of Agriculture (USDA), and the Public Health Service Policy on the Humane Care and Use of Laboratory Animals, overseen by the NIH Office of Laboratory Animal Welfare (OLAW).

## Results

### Ocular Disease Phenotypes of the OD4-CJ994 Recombinants

**Fig 1:**
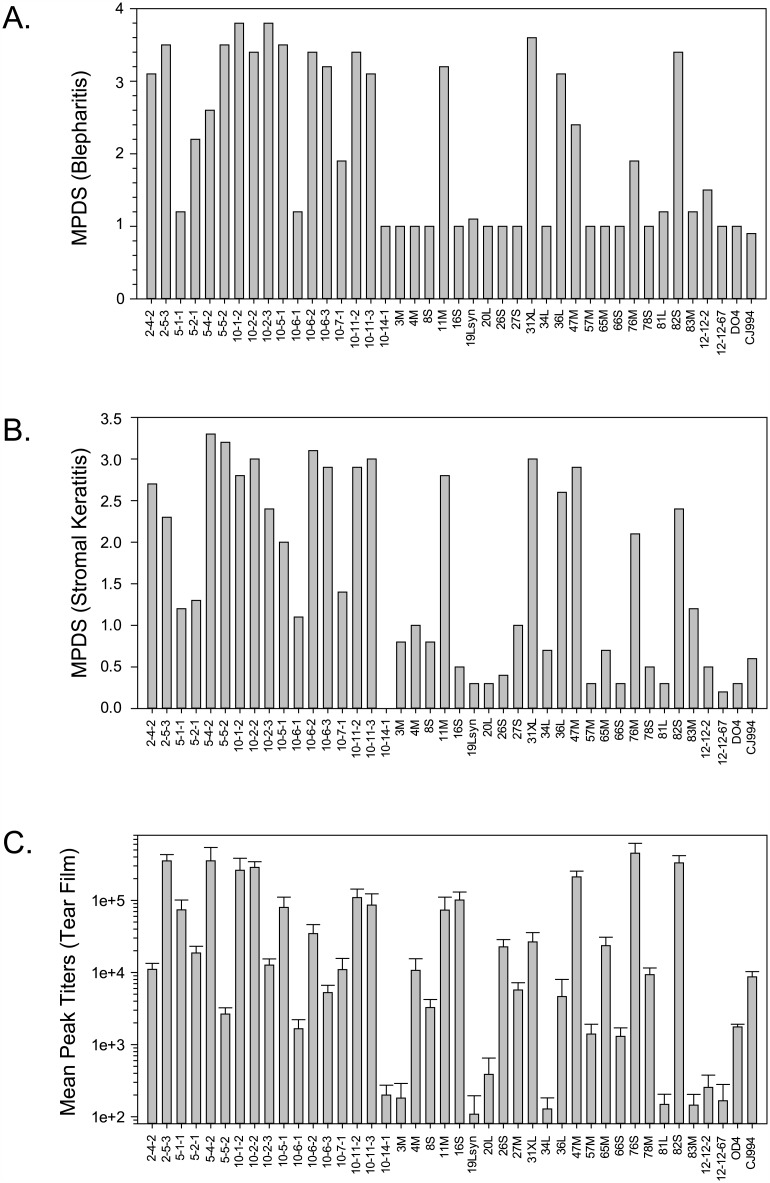
Mean peak disease scores (MPDS) and mean peak tear film titers of the OD4-CJ994 recombinants. (A) Blepharitis MPDS scores of the 40 OD4-CJ994 recombinants. (B) Stromal keratitis MPDS scores of the 40 OD4-CJ994 recombinants. (C) Mean peak tear film titers ± SEM of the 40 OD4-CJ994 recombinants.

We previously published the sequences and recombination maps of 40 OD4-CJ994 recombinants [34]. To obtain quantitative disease scores, 10 mice per group were infected by corneal scarification with each recombinant strain, and the severity of blepharitis and stromal keratitis was determined. Although we had previously identified virulence phenotypes for the *in vivo* recombinants [30] we re-determined them for the current study to insure consistency across the dataset. The mean peak disease scores (MPDS) for each recombinant are shown in Fig 1. A range of MPDS scores was observed, ranging from the low pathogenic recombinant 10-14-1 (blepharitis 1; stromal keratitis 0) to high pathogenic recombinants like 10-2-2 (blepharitis 3.4; stromal keratitis 3). Notably, the *in vivo* derived recombinant strains exhibited higher MPDS scores than the *in vitro* derived recombinants. The mean blepharitis MPDS score of the *in vivo* derived strains was 2.81 versus 1.54 for the *in vitro* derived strains, which was significantly different (*p* = <0.001). The mean stromal keratitis MPDS scores were 2.2 for the *in vivo* derived recombinant strains, compared to 1.11 for the *in vitro* strains. These differences were also statistically significant (*p* = <0.001). We also observed mortality with the 2-4-2, 2-5-3, 10-1-2, 10-2-3, 10-5-1, 10-6-2, 10-6-3, 10-11-2, 11M, 31XL, 36L, and 82S recombinants. We do not have enough power with the current data set to be able to identify mortality determinants using the vQTLmap analysis.

### Ability to Replicate Is Associated with Increased Virulence

**Fig 2:**
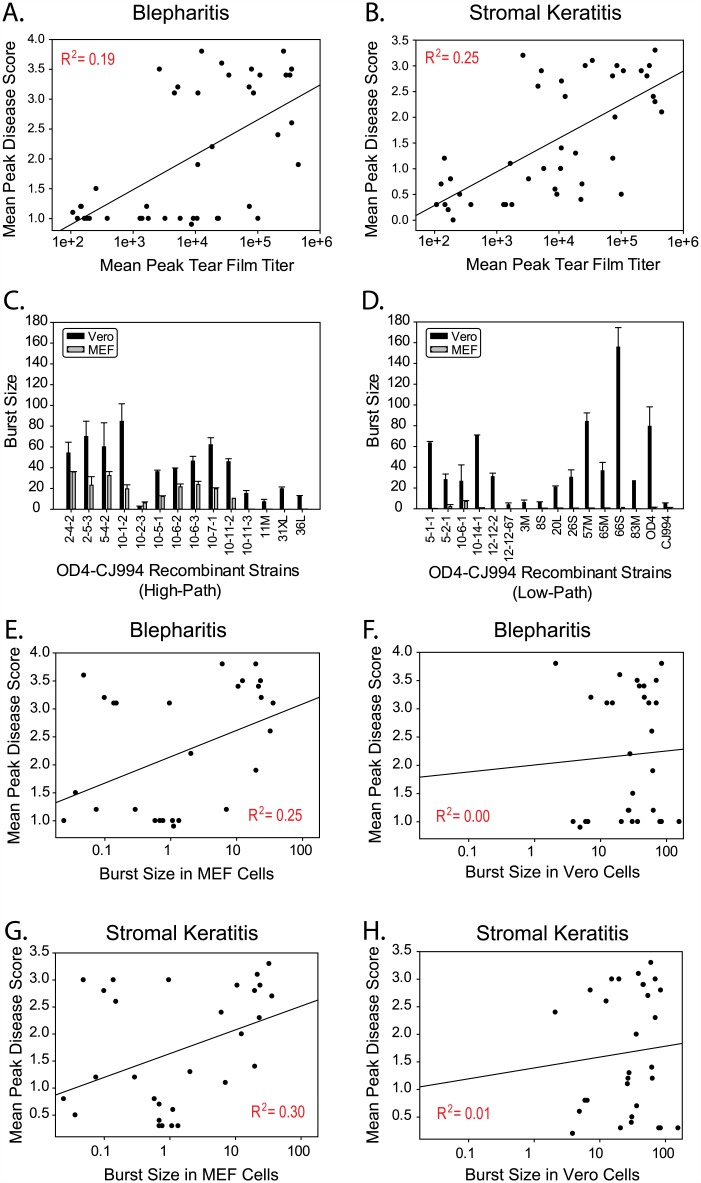
MPDS vs. mean peak titer scatter plots of the OD4-CJ994 recombinants and one step growth curves of seven high pathogenic and nine low pathogenic recombinants. Scatterplots of mean peak disease scores (MPDS) for blepharitis (A) and stromal keratitis (B) versus mean peak tear film titers of the 40 OD4-CJ994 recombinants. The R^2^ values indicate the strength of the associations as determined by linear regression. (C) One step growth curve titers of 14 high pathogenic OD4-CJ994 recombinants in Vero cells (black) and mouse embryonic fibroblast cells (MEF; gray). (D) One step growth curve titers of 14 low pathogenic OD4-CJ994 recombinants as well as the OD4 and CJ994 parentals in Vero cells (black) and MEF cells (gray). (E) Scatterplot of blepharitis MPDS scores vs. MEF replication burst size with R^2^ linear regression values. (F) Scatterplot of blepharitis MPDS scores vs. Vero cell replication burst size with R^2^ linear regression values. (G) Scatterplot of stromal keratitis MPDS scores vs. MEF replication burst size with R^2^ linear regression values. (H) Scatterplot of stromal keratitis MPDS scores vs. Vero cell replication burst size with R^2^ linear regression values.

To assess the ability of each OD4-CJ994 recombinant to replicate *in vivo*, corneal tear samples were taken on days 1–7 post-infection, and the mean peak titers for each recombinant are shown in Fig 1C. There was at least a 3-log difference in the mean peak titers, which ranged from 1.48 x 10^2^ pfu/mL for recombinant 81L to 4.5 x 10^5^ pfu/mL for the 76M recombinant. The average mean peak titer for the *in vivo* strains was 1 x 10^5^ pfu/mL versus 5.5 x 10^4^ pfu/mL for *in vitro* derived recombinants and was statistically significant (*p* = 0.023). Linear regression analysis of MPDS scores versus mean peak tear film titers suggest that there may be two separate populations for the blepharitis and stromal keratitis phenotypes (Fig 2A and 2B). The results also suggest that ocular disease severity and ability to replicate *in vivo* may be linked, with the majority of low pathogenic or high pathogenic recombinants associating together. There were however, several low pathogenic recombinants such as 4M, 16S, and 65M that replicated to levels similar to that of the high pathogenic strains (Figs 1 and 2A).

Based on these results, we reasoned that replication of the low and high pathogenic recombinant strains would be similar in the highly permissive Vero cells, but that the low pathogenic recombinants would replicate less well in the presumably less permissive mouse embryonic fibroblast (MEF) cells. We selected 14 high pathogenic and 14 low pathogenic recombinants for testing and determined titers at 18 hours post-infection at an MOI of 1. The results show that the 14 high pathogenic and 14 low pathogenic strains burst sizes were similar in Vero cells (Fig 2C and 2D), but there were several outliers. However in MEF cells, most of the low pathogenic strain had burst sizes close to zero (Fig 2B and 2C). It should be noted that the high pathogenic strains titers were also reduced in MEF cells compared to Vero cells (Fig 2B), however the burst sizes were generally higher. To further examine the differences of one step growth curves in Vero versus MEF cells, we generated scatterplots of mean peak disease scores versus burst size for blepharitis and stromal keratitis. Fig 2E shows a weak correlation between blepharitis MPDS and MEF burst size (R^2^ = 0.25), while in Vero cells there was no correlation (R^2^ = 0.00; Fig 2F). There was also a weak correlation between stromal keratitis MPDS versus MEF burst size (R^2^ = 0.30; Fig 2G), while no correlation was observed in Vero cells (R^2^ = 0.01; Fig 2H). These results are similar to the *in vivo* titer results and suggest that the ability to replicate in the host is an important driver of virulence, although it is not the only factor since the correlative R^2^ values were relatively weak.

### vQTLmap Analysis

Three machine learning methods, Random Forest, Lasso, and Ridge regression, were evaluated to determine which provided the highest predictive accuracy when mapping OD4-CJ994 recombinant genotypes to ocular phenotypes. The recombinant genotypes were represented using 491 haplotype blocks that were derived from a multiple sequence alignment of the parental and recombinant genomes. We used leave-one-out cross validation to assess the predictive accuracy of the three methods for the three phenotypes.

**Fig 3:**
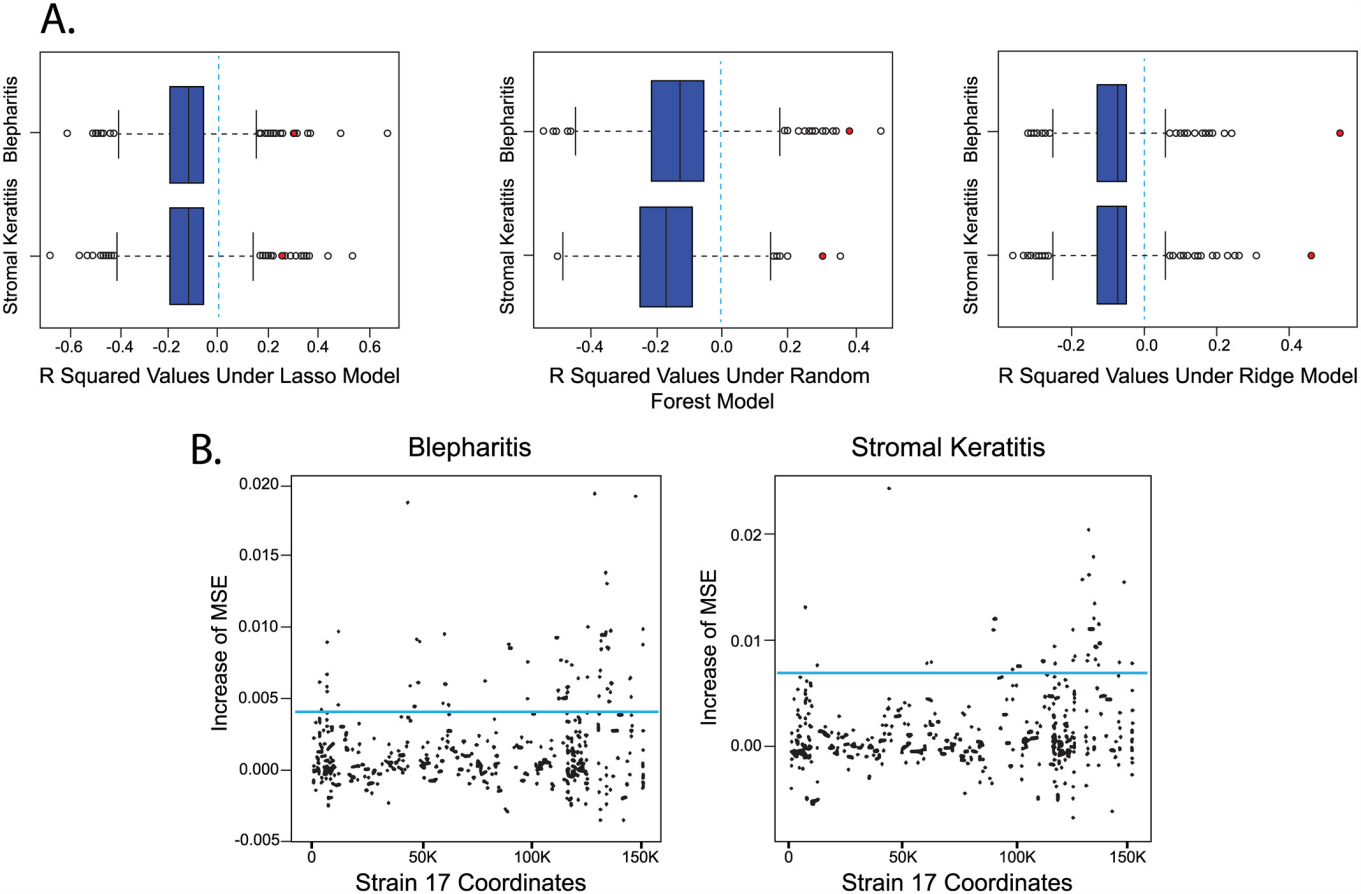
Evaluation of learned vQTLmap models mapping 40 OD4-CJ994 recombinant genotypes to ocular phenotypes. (A) The results of cross-validated predictions for blepharitis and stromal keratitis using learned Lasso, Random Forest, and Ridge regression models. The red circles indicate the R^2^ values for models learned from the actual data, whereas the blue box plots show the R^2^ values for models learned from 1,000 permuted datasets. The vertical blue dotted lines indicate the R^2^ values for the non-learned baseline. (B) Association between 491 loci and the two phenotypes as determined by the Ridge regression models. The horizontal axis represents coordinates in the HSV-1 strain 17 reference genome, and the vertical axis represents the change in the mean squared error (MSE) of predicted phenotypes when the values for a given locus are permuted. The horizontal blue lines indicate the thresholds for associations to be considered significant.

The accuracies of the cross-validated predictions for blepharitis and stromal keratitis using the three learning methods are shown as red points in Fig 3A. To determine whether these R^2^ values represent statistically significant predictability, we used a Monte Carlo methodology in which the cross-validation procedure was repeated 1,000 times with the measured phenotypes randomly shuffled and then reassigned to the haplotype feature vectors. The box plots in Fig 3A show the resulting R^2^ values for models learned from the randomized genotype-phenotype data sets. These results indicate that the learned models have statistically significant predictive power for the blepharitis and stromal keratitis phenotypes.

Since the R^2^ values for ocular disease phenotypes were highest using Ridge regression, we use these models for the subsequent QTL based virulence association analysis (vQTLmap). Fig 3B shows Manhattan plots depicting the association between the 491 loci (haplotype blocks; S1 Table) and the two phenotypes as determined by the Ridge regression models. The horizontal axis represents coordinates in the HSV-1 strain 17 reference genome, and the vertical axis represents the change in the mean squared error (MSE) of predicted phenotypes when the values for a given locus are permuted. The horizontal blue lines indicate the thresholds used for the associations to be considered significant.

### Detection of Features Associated with Ocular Disease Phenotypes

Following the vQTLmap analysis, phenotypically important features from the learned models were extracted, with a full list found in S2 Table. The ocular phenotype with the highest number of considered associations was blepharitis followed by stromal keratitis (S2 Table). A few of the detected associations may be false positives as they occur in poor quality sequence regions (e.g. 126,825 IRS, 6,456 TRL, ICP0 S305G). Because of the unreliability of the sequence in the indicated regions, these features were not considered further. A collection of highly scoring, notable associations are listed in Table 2. A few notable intergenic and promoter regions were detected, including intergenic regions between UL21 and UL22 (blepharitis and stromal keratitis), and UL3 and UL4 (blepharitis). Several features mapped to either 5’ or 3’ UTR regions of genes, including UL3, US1, UL30, and US3. Highly scoring features encoding synonymous variations were found in the UL25 (blepharitis), UL40 (blepharitis and stromal keratitis), and US2 (blepharitis, stromal keratitis) genes. Notable associations encoding amino acid variations were found (where the OD4 SNP:amino acid postion:CJ994 SNP) in the ICP4 (M454L; blepharitis and stromal keratitis), US3 (T52P; blepharitis and stromal keratitis), gG (K96E, V113F, G117E, V119D, P131S, G133D, S152I, Q163R; blepharitis and stromal keratitis), and VHS (L374R, N384S, fs*475P; stromal keratitis) proteins.

**Table 2:** Highly scoring associations detected by QTL analysis of blepharitis and stromal keratitis with the associated amino acid encoding variations.

Blepharitis			Stromal Keratitis		
Strain 17 coordinate	I.O.M.E^&^	Gene: SNP variation	Strain 17 coordinate	I.O.M.E^&^	Gene: SNP variation
129,769/148,464	0.0194 0.0192	**ICP4:** M454L*	43,740	0.0247	**I.G. UL21/UL22** ^#^
43,740	0.0188	**I.G. UL21/UL22** ^§^	134,773	0.0181	**US2:** no a.a. change*
134,773	0.0137	**US2:** no a.a. change*	132,601–132,781	0.0164	**US1 5’UTR-US1:** S45T, E46D^#^
135,290	0.0129	**US3:** no a.a. change^§^	129,769/ 148,464	0.0159	**ICP4:** M454L*
137,023–137,225	0.0096	**US4 (gG):** K96E, V113F, G117E, V119D, P131S, G133D, S152I, Q163R^§^	135,290	0.0136	**US3:** no a.a. change*
11,676	0.0095	**UL3:** 3’ UTR#	134,955	0.0122	**US2 gene** ^§^
60,771	0.0095	**UL29 (ICP8):** A428V*	90,849–91,546	0.0121	**UL40-UL41 (VHS):** L374R, N384S, fs475P*
132947–134598	0.0093	**US1-US2:** A102T, S103A, C116Y, A215T^#^	137,023–137,225	0.0116	**US4 (gG):** K96E, V113F, G117E, V119D, P131S, G133D, S152I, Q163R^§^
112,472–113,150	0.0091	**UL53 (gK):** S305L^§^	132,947–134,598	0.0112	**US1-US2:** A102T, S103A, C116Y, A215T^§^
47,877	0.0090	**UL24:** no a.a. change^#^	90,342–90,597	0.0110	**UL40 (RR2):** no a.a. change*
49,289	0.0088	**UL25:** no a.a. change^#^	135375	0.0095	**US3:** T52P^§^
90,342–90,597	0.0086	**UL40 (RR2):** no a.a. change*			
135,375	0.0084	**US3:** T52P^§^			
90,849–91,546	0.0084	**UL40-UL41 (VHS):** L374R, N384S, fs475P*			
114,619–114,707	0.0075	**UL54 (ICP27):** I325T*			
48309–48734	0.0059	**UL24-UL/25:** M191I, A210V, V231L^#^			
116093–117031	0.0048	**I.G UL55/56-UL56 promoter:** (UL56) S58L, V121A, P143H,G213W^§^			

*Annotation detected in Random Forest, Lasso, and Ridge regression models

^§^Annotation detected in Ridge regression and either the Random Forest or Lasso models

^#^Annotation detected in Ridge regression only

^&^I.O.M.E = increase of mean squared error

### Mapping Amino Acid Encoding Associations to their Corresponding Proteins

**Fig 4:**
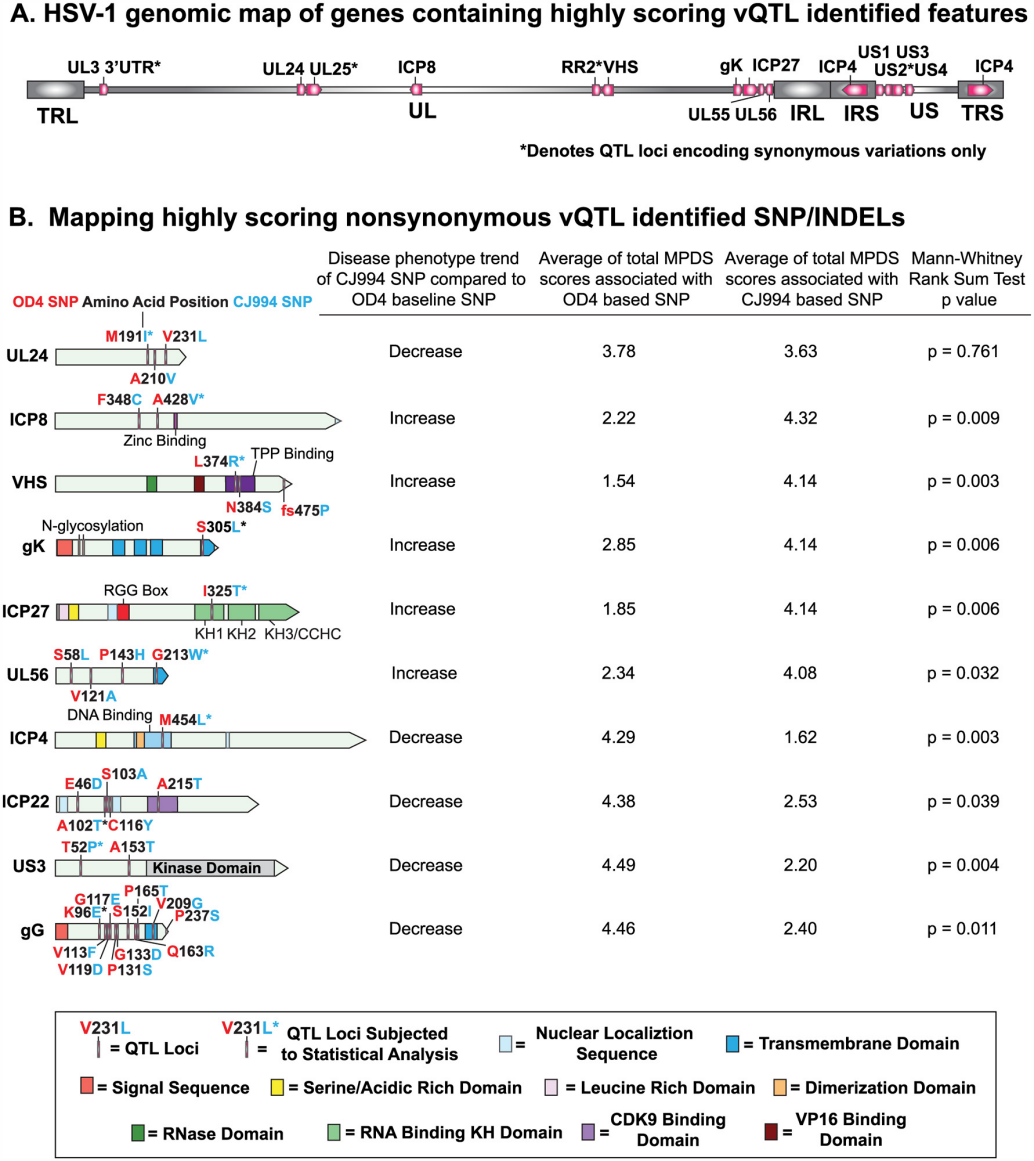
Map of high scoring nonsynonymous vQTLmap detected SNP/INDELs and statistical analysis of ocular disease trends. (A) HSV-1 genomic map of genes containing high scoring vQTLmap identified features (pink). (B) Mapping of nonsynonymous highly scoring features identified by vQTLmap analysis to their corresponding proteins. Functional domains and motifs have also been mapped in each protein if applicable with the key at the bottom of the Fig. The trends of ocular disease associated with vQTLmap identified SNPs are found to the right of the maps. The parental strain OD4 was designated as the baseline with the disease trend associated with the CJ994 SNP variation to the right of each protein map. The average of the sum of the MPDS scores associated with OD4 and CJ994 have also been included. Mann-Whitney rank sum tests were performed on the MPDS scores of the recombinants containing OD4 versus CJ994 variants, with the resulting *p*-values shown at the end of each row.

We next mapped the highly scoring vQTLmap identified nonsynonymous SNPs to their corresponding proteins to determine if any of the detected variations occur in known functional sites or motifs. First, the vQTLmap identified genes were mapped to the HSV-1 genome (Fig 4A). Fig 4B shows the mapped vQTLmap features. Several associations mapped to known functional motifs, such as the VHS (UL41) identified SNPs L374R and N384S, which map to the tristetraprolin binding region. In ICP4 (RS1), the M454L feature maps to the DNA binding II domain, and the I325T association in ICP27 (UL54) maps to the RNA binding KH1 domain. There were several detected features which are consistent with possible serine/threonine phosphorylation sites in known phosphorylated proteins; ICP27 (I325T), ICP22 (A102T, S103A and A215T), US3 (T52P and A153T). Phenotypically meaningful variations in the gK (S305L) and UL56 (G213W) proteins mapped to transmembrane domains. The UL24 and ICP8 detected associations did not map to any known functional domains.

### Phenotypic Trends and Statistical Analysis of Selected Nonsynonymous Associations

Ocular disease trends of the identified vQTLmap associations were determined by setting the OD4 variation as the baseline. In Fig 4B the ocular disease phenotype trends for nine of the proteins identified in vQTLmap are shown. The average of total MPDS scores associated with the OD4 and CJ994 parental SNPs, along with Mann-Whitney rank sum test *p*-values are also shown (Fig 4B; S4 Table). Decreasing disease trends associated with CJ994 SNPs compared to the OD4 baseline SNPs were observed in UL24, ICP4, ICP22, US3, and gG. All of the associations that correlated with a downward trend in virulence were statistically significant, with the exception of UL24 which was not considered for further analysis. The vQTLmap identified features in ICP8, VHS, gK, ICP27, and UL56 were associated with an increase in virulence compared to the strain OD4 baseline SNPs, and all were statistically significant.

### Virulence Gene Network

**Fig 5:**
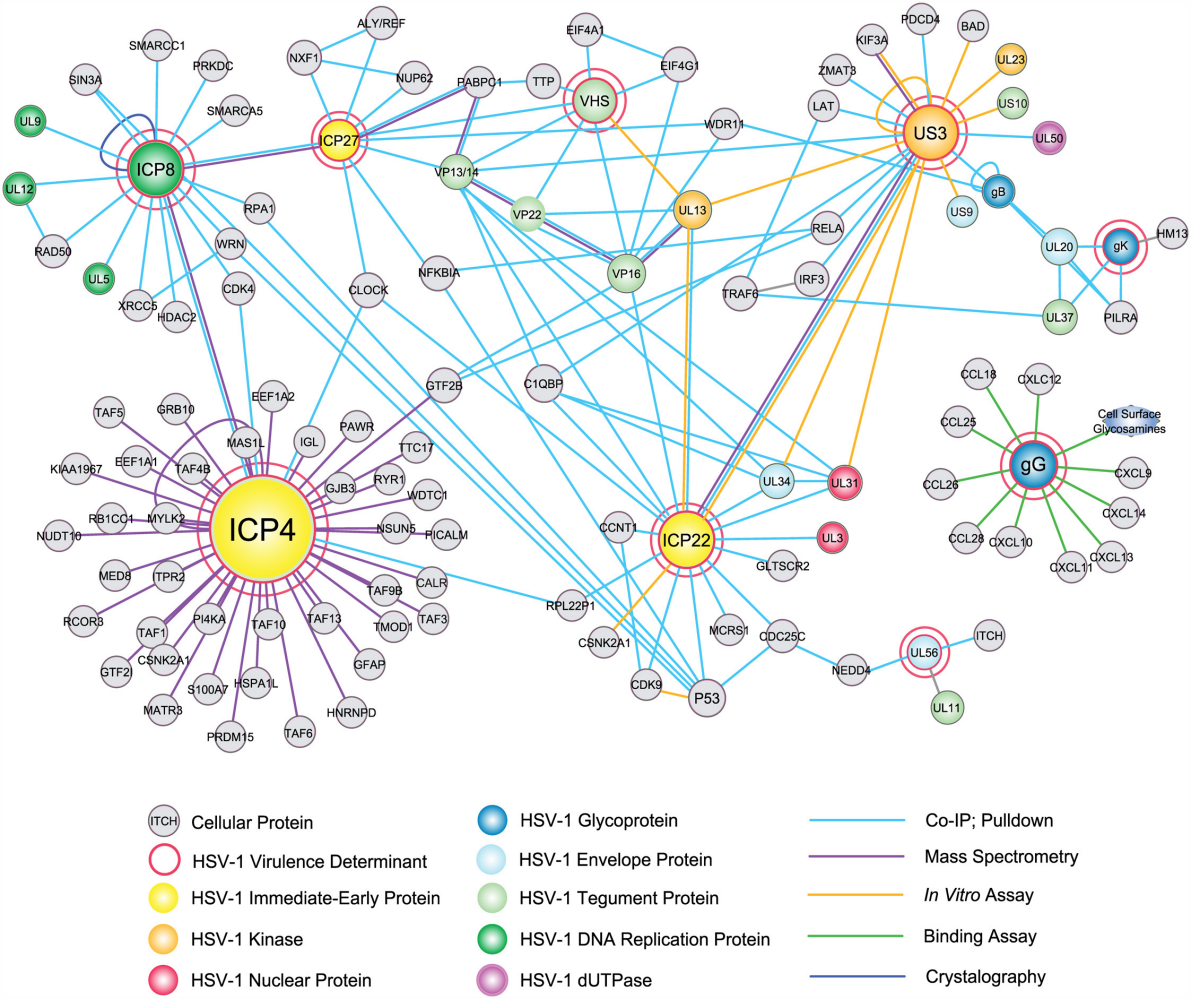
Protein-protein interaction network of high scoring vQTLmap identified virulence proteins. A key is provided near the bottom of the Fig. The size of the nodes corresponds to the number of interactions. The network was produced by literature search using the GADGET tool, and the display was generated using Cytoscape 3.2.0.

To aid the interpretation of the vQTLmap data, a protein-protein interaction network was constructed (Fig 5; S3 Table). All of the identified major virulence proteins had multiple protein-protein interactions. Several of the major virulence proteins detected in this study have been shown to interact with each other; including ICP4 and ICP8, ICP8 and ICP27, ICP27 and VHS, ICP22 and US3. Also, with the exception of gG, the remaining virulence genes were connected through secondary or tertiary interactions, e.g. US3-VP13/14-ICP27, ICP4-ICP8-ICP27, and ICP4-CLOCK-ICP22. Importantly the ICP27, US3, and gG proteins directly interact with host immune system proteins. Glycoprotein G directly interacts with several chemokines, ICP27 binds IκBα (NFKBIA), and US3 hyperphosphorylates both IR3 and p65/RELA. The significant virulence determinants were further categorized into three functions groups; transcription, virion assembly/egress, and immunomodulation (Table 3). Several of the proteins reside in overlapping functional groups, for example ICP22 plays a function in both transcription and virion assembly/egress, and ICP27 plays a role in transcription as well immunomodulation. Additionally, ICP8 was the only β class gene that was identified in the virulence determinant network.

**Table 3:** Functional groups of viral genes identified by vQTLmap.

Functional Group	Virulence Gene	Essential (E) or Dispensable (D)	Kinetic Class
**Transcription**	ICP8	E	β
	VHS	D	γ1
	ICP27	E	α
	ICP4	E	α
	ICP22	D	α
**Virion Egress**	gK	E	γ2
	UL56	D	γ2
	ICP22	D	α
	US3	D	γ1
**Immunomodulation**	VHS	D	γ1
	ICP27	E	α
	US3	E	γ1
	gG	D	γ1

## Discussion

Previous linkage studies examining virulence in HSV-1 have focused on identifying host factors influencing the severity of ocular disease and herpes simplex encephalitis in either mice or rats [11, 15, 23, 24, 45]. To this point, no attempts have been made to implement QTL analysis to identify virulence determinants in HSV-1. In the current study, we describe a novel method for performing virulence QTL mapping (vQTLmap) for viral virulence determinant discovery in HSV-1 ocular infections. The results demonstrated the i) validity of linkage analysis for HSV-1, ii) the Ridge regression model was the most predictive learner for predicting phenotype scores with the current dataset, iii) multiple vQTLmap features were associated with the blepharitis and stromal keratitis phenotypes, and iv) several vQTLmap identified features mapped to important functional areas of proteins, i.e. ICP4 M454L. Together this data lays the groundwork for future virulence studies and methods that may also be applied to other viruses as well as for the analysis of epistatic interactions.

### HSV-1 OD4-CJ994 Recombinant Phenotypes

We have previously shown that 17 HSV-1 OD4-CJ994 recombinants exhibit a wide range of ocular disease phenotypes in mice [30]. It is also notable that we were able to generate virulent recombinants from the two low virulence parental strains, suggesting that different combinations of parental genes are involved in determining virulence. Following ocular virulence characterization in our mouse ocular model, the *in vivo* derived strains were statistically more virulent and had significantly higher mean peak tear film titers than the *in vitro* derived strains. We hypothesize that selective pressure in the mouse cornea results in recombinants that are better adapted for replication *in vivo*, unlike the *in vitro* derived recombinants which were subjected to different selection pressures. The *in vitro* recombinants were generated in Vero cells which are highly permissive, do not synthesize interferon, and may have other innate defects that contribute to their permissiveness (Fig 2C and 2D). If we had generated recombinants in MEF cells, we might have selected for different traits. Likewise, the linear regression scatter plots and growth studies of high pathogenic and low pathogenic recombinants in mouse embryonic fibroblast (MEF) cells (Fig 2) suggest that virulence is a function of ability to replicate and that replication may be an important driver of virulence. It should be noted that the R^2^ values for the linear regression analyses of mean peak titer versus phenotypic disease were low, with several low pathogenic strains replicating to high titers (Figs 1 and 2), suggesting that additional factors beyond replication efficiency contribute to virulence. Finding a correlation between virulence and replication in HSV-1 is not unexpected, as similar phenomena have been observed in poliovirus 1 [46], vesicular stomatitis virus [47], and Marek’s disease virus, another alphaherpesvirus [48]. Based on this finding, determining the viral replication potential of HSV-1 strains in MEF cells or another restrictive cell line may be a rapid method of estimating HSV-1 virulence.

### Virulence Determinant Study Comparison

**Fig 6:**
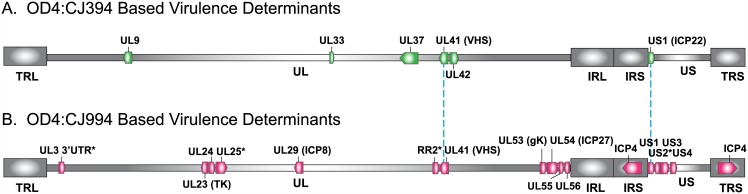
Comparison of HSV-1 OD4:CJ394 recombinant versus OD4:CJ994 recombinant based virulence determinants. (A) Map of virulence determinants derived from OD4:CJ394 based marker transfer virulence studies [32]. (B) Map of OD4:CJ994 based virulence determinants derived from the vQTLmap method described in the current study. The blue dotted line highlights common virulence determinants between the OD4:CJ394 and OD4:CJ994 virulence studies.

Traditionally, studies on the genetics of virulence have relied on isolating and characterizing a naturally occurring viral strain with altered virulence properties, genetically engineering either deletions or point mutations, or using marker transfer methods to exchange genes between strains. A previous study of ours used marker transfer/infection to exchange different combinations of genes from a moderately virulent strain (CJ394) into the attenuated HSV-1 strain OD4, and found that virulence phenotypes in mice was dependent on the combination of genes that were transferred [32], suggesting epistatic interactions play a role in virulence. We found that two mutations in the ICP22 protein (US1) needed to be co-inherited for the virulence phenotype supporting a role for epistasis in determining virulence [32]. Additionally, the study identified several virulence determinants including UL9, UL33, UL37, UL41 (VHS), UL42 and US1 (ICP22) (Fig 6a). When the current vQTLmap study which is based on OD4:CJ994 recombinants and the previous study (OD4:CJ394) are compared, only the UL41 and US1 genes overlapped as virulence determinants (Fig 6). This lack of virulence gene overlap between the two HSV-1 strain combinations suggests that complex epistatic interactions are involved in determining ocular virulence phenotypes.

### Virulence Determinant Functional Groups

While the majority of downstream analysis of the vQTLmap features focused on amino acid encoding SNPs, intergenic, 5’ and 3’ gene UTR, and promoter virulence associations were also identified. The only intergenic feature identified was an INDEL located between the UL21 and UL22 genes. There are no annotated regulatory elements identified in this region, and we are uncertain if the phenotypic importance of the INDEL is due to an uncharacterized regulatory element or the influence of SNPs in the surrounding genes. Features in the 5’ and 3’ UTR regions of UL3, UL30, US1, US2, and US3 were also detected by vQTLmap, and as with the intergenic region described above, we are not certain if the features contain unknown regulatory sequences or are influenced by SNPs in the surrounding genes. Features mapping to the UL56, ICP4, and IE 4/5 promoters were also detected. Examination of these promoter regions did not reveal a gain or loss of any notable transcription factor binding sites, so the importance of these regions as virulence determinants remains unclear.

The vQTLmap analysis identified several novel amino acid encoding feature SNPs, including ICP8, VHS, gK, ICP27, UL56, ICP4, US1, US3, and gG (Table 2, Fig 4). When these genes were examined in the protein: protein interaction network (Fig 5), we were able to classify the genes into three functional clusters (Table 3); transcription, virion assembly-egress, and immunomodulation. The ICP8, VHS, ICP27, ICP4, and ICP22 proteins are associated with various components of viral transcription [49–51]. While we are uncertain as to exactly how the detected virulence associated SNPs in each of these proteins could be affecting transcription, there are several SNPs warranting closer inspection. In UL41-VHS, Two of the variations (L374R/N384S) map to the tristetraprolin (TPP) binding domain of VHS. Tristetraprolin has been shown to recruit VHS to AU rich elements in stress mRNAs for subsequent cleavage [52], and it is possible that the vQTLmap variations may negatively affect the recruitment of VHS by TPP. Additionally, a frameshift at position 475 associated with a reduction in virulence extends the VHS protein by 47 amino acids and may adversely alter VHS functionality. The ICP27 protein contained one SNP (I325T). ICP27 appears to mediate VHS degradation of mRNA [53, 54] and is a component of the HSV-1 transcriptome complex [51]. The vQTLmap feature (I325T) mapped to the KH1 RNA binding domain of ICP27. ELM motif prediction found that the T325 residue is part a putative GSK3 phosphorylation motif, and it should be noted that the loss of the threonine is associated with reduction in virulence. Regulation of KH domain binding to mRNA has been shown to be regulated by phosphorylation [55], and it is possible that phosphorylation of T325 could be playing a similar function. In ICP4, the M454L variation maps to a methionine residue in the DNA binding domain 2 of ICP4 which is highly conserved across alphaherpesvirinae [56]. We hypothesize that the leucine at position 454 is negatively affecting DNA binding, and thus reducing expression of viral late genes, as well regulatory binding to target promoters. Support for this hypothesis comes from mutational studies of the ICP4 DNA binding region residues 442–485 showing that several mutations in DNA binding domain II resulted in reduced DNA binding phenotypes [57].

Glycoprotein K (gK), UL56, US1 and US3 have been implicated in virion egress [58–63]. Notably, UL56 contains three SNPs, with one G213W occurring near the N-terminal putative transmembrane domain. Transmembrane domain analysis of the UL56 protein, predicts a reduction of transmembrane potential with a glycine at position 213, as compared to the hydrophobic tryptophan residue, and may therefore affect membrane anchoring stability or the ability to interact with other membrane bound proteins. Similarly, the S305L SNP in gK maps to the C-terminal transmembrane domain, and may affect membrane anchoring.

The immunomodulatory proteins VHS, ICP27, US3 and gG appear to be converging on adaptive immunity. The US3 serine/threonine kinase contained five separate vQTLmap features for the blepharitis and stromal keratitis phenotypes, but only two, T52P and A153T, encoded a nonsynonymous variation (Table 2; S2 Table). US3 is a multifunctional kinase and neurovirulence determinant [64, 65] that has immunomodulatory activity by hyperphosphorylating IRF3 and p65/RELA [66, 67], inhibiting T cell signaling through confining the host T-cell activator component LAT [68], as well as interacting with PDCD4 to block apoptosis [69]. The function of the vQTLmap identified T52P and A153T variations is unclear as they occur outside of the US3 kinase domain. The T52P and A153T variations instead map to a putative disordered region, and may affect the ability of US3 to bind to interaction partners. The vQTLmap analysis identified a 202 bp feature containing the K96E/V113F/G117E/V119D/P131S/G133D/S152I/Q163R variations which mapped to gG’s extracellular domain (Fig 4). A second lower scoring 575 bp feature also mapped to gG and included the P165T/V209G/P237S variations. The V209G variation mapped to gG’s transmembrane region and the P237S variation mapped to the cytoplasmic tail. Recently, glycoprotein G has been shown to bind the glycosaminoglycan-binding domain of several chemokines including CCL18, CCL28, CXCL9, and CXCL14 [70]. The significance of how the vQTLmap amino acid variations in gG may affect virulence is uncertain, however, it is not unreasonable to hypothesize that at least one of detected variations may affect chemokine binding efficiency and affect innate or adaptive immune responses.

While the significance of the SNPs in VHS, ICP27, and US3 are not yet clear, these three proteins affect the interferon response. VHS inhibits Stat-1 phosphorylation and prevents formation of the Stat-1/2-p48 complex [71, 72], reducing interferon levels. ICP27 inhibits IRF-3 activation, blocking interferon expression [73]. NF-κβ activity is reduced by both ICP27 and US3. The ICP27 protein physically interacts and stabilizes IκBα, lowering NF-κβ activity, and US3 hyperphosphorylates p65/RELA reducing NF-κβ activation [66, 74]. US3 also hyperphosphorylates IRF3, which in turn blocks IRF3 nuclear translocation and IFN-β activation.

### Summary

In summary this is the first report using QTL based analysis of HSV-1 for virulence gene discovery. The analysis successfully detected previous and unidentified virulence genes and novel associated SNPs. This data lays the groundwork for future virulence studies and methods that may also be applied to other viruses.

## Supporting Information

S1 Table Table listing the 491 loci (haplotype blocks) used in the vQTLmap regression models. The first column of the table shows the name of each feature, consisting of ‘X’ followed by the corresponding coordinates in the multiple sequence alignment. Features representing haplotype blocks of multiple positions are named as ‘X’ followed by the first and last positions of the variations that were merged, with the number of merged variations in parentheses. The second column shows the specific positions of the haplotype feature variations. The third and fourth columns show the bases, insertions or deletions that the parental strains, OD4 and CJ994, have at the positions indicated in the second column.
(XLSX)

S2 Table List of the phenotypically meaningful features identified for each of the three vQTLmap regression models. The loci coordinates beginning with and “X” are based on the multiple sequence alignment, and are found under the Lasso, Random Forest, and Ridge regression model columns. The Ridge regression model had the highest R^2^ values for both blepharitis and stromal keratitis. The increase of mean squared error values for the Ridge model is shown in the “Increase of MSE” columns. The Ridge model identified features have loci coordinates normalized to HSV-1 Strain 17, and are found in the “Strain 17 Coordinates column”. Columns describing the corresponding genes are located in the adjacent “Gene” column.
(XLSX)

S3 Table vQTLmap virulence protein-protein interaction library. The viral genes, protein, interaction partners, Entrez IDs, line of evidence and literature references are placed in separate columns.
(XLSX)

S4 Table Table of MPDS and SNP information for each of the major vQTLmap identified features. Each major nonsynonymous vQTLmap locus has a separate page in the EXCEL file. For each page the mean peak disease scores (MPDS) for blepharitis and stromal keratitis, as well as the sum of these values for each of the 40 recombinants are shown in separate columns. The column containing the SNP nucleotides associated with each recombinant strain is adjacent to the “sum of MPDS” column. The OD4 and CJ994 associated SNPs are placed in separate columns. The mean value of the sum of the MPDS scores for each of the OD4 and CJ994 SNPs, as well as the Mann-Whitney rank sum test *p*-values are located in the lower right of the table.
(XLSX)
